# Novel insights into non-alcoholic fatty liver disease and dementia: insulin resistance, hyperammonemia, gut dysbiosis, vascular impairment, and inflammation

**DOI:** 10.1186/s13578-022-00836-0

**Published:** 2022-06-28

**Authors:** So Yeong Cheon, Juhyun Song

**Affiliations:** 1 Department of Biotechnology, College of Biomedical & Health Science, Konkuk University, Chungju, Republic of Korea; 2 Department of Anatomy, Chonnam National University Medical School, Hwasun, Jeollanam-do 58128 Republic of Korea

**Keywords:** Non-alcoholic fatty liver disease, Dementia, Alzheimer’s dementia, Vascular dementia, Diabetes-induced dementia

## Abstract

Non-alcoholic fatty liver disease (NAFLD) is a metabolic disease characterized by multiple pathologies. The progression of dementia with NAFLD may be affected by various risk factors, including brain insulin resistance, cerebrovascular dysfunction, gut dysbiosis, and neuroinflammation. Many recent studies have focused on the increasing prevalence of dementia in patients with NAFLD. Dementia is characterized by cognitive and memory deficits and has diverse subtypes, including vascular dementia, Alzheimer’s dementia, and diabetes mellitus-induced dementia. Considering the common pathological features of NAFLD and dementia, further studies on the association between them are needed to find appropriate therapeutic solutions for diseases. This review summarizes the common pathological characteristics and mechanisms of NAFLD and dementia. Additionally, it describes recent evidence on association between NAFLD and dementia progression and provides novel perspectives with regard to the treatment of patients with dementia secondary to NAFLD.

## Background

Non-alcoholic fatty liver disease (NAFLD), one of the most common causes of liver disorders worldwide, is a progressive chronic disease and a metabolic-associated fatty liver disease [1]. It ranges from fatty liver to liver cirrhosis, which can ultimately lead to hepatocellular carcinoma [2]. Non-alcoholic fatty liver often progresses to severe non-alcoholic steatohepatitis (NASH), liver fibrosis, and liver cirrhosis [2]. Lifestyle, eating habits, and genetic background all contribute to the morbidity and mortality of NAFLD [3, 4]. In particular, metabolic disturbances (e.g., abnormal uptake of hepatic fatty acid, imbalanced lipid synthesis, and obesity) are known to contribute to fatty liver via excessive triglyceride accumulation in hepatocytes [5]. NAFLD is affected by common risk factors as other metabolic disorders, such as type 2 diabetes mellitus (T2DM), chronic kidney disease (CKD), and cardiovascular disease (CVD) [6, 7]. Moreover, NAFLD is a risk factor itself for other metabolic diseases [6, 8]. According to an epidemiological study, the prevalence of NAFLD in patients with diabetes is estimated to be > 50% [3]. Patients with fatty liver showed a high occurrence of coronary artery disease [9] and a high risk for CVD [10]. In addition, patients with NAFLD had a high prevalence of CKD compared with patients without NAFLD [8].

**Fig. 1:**
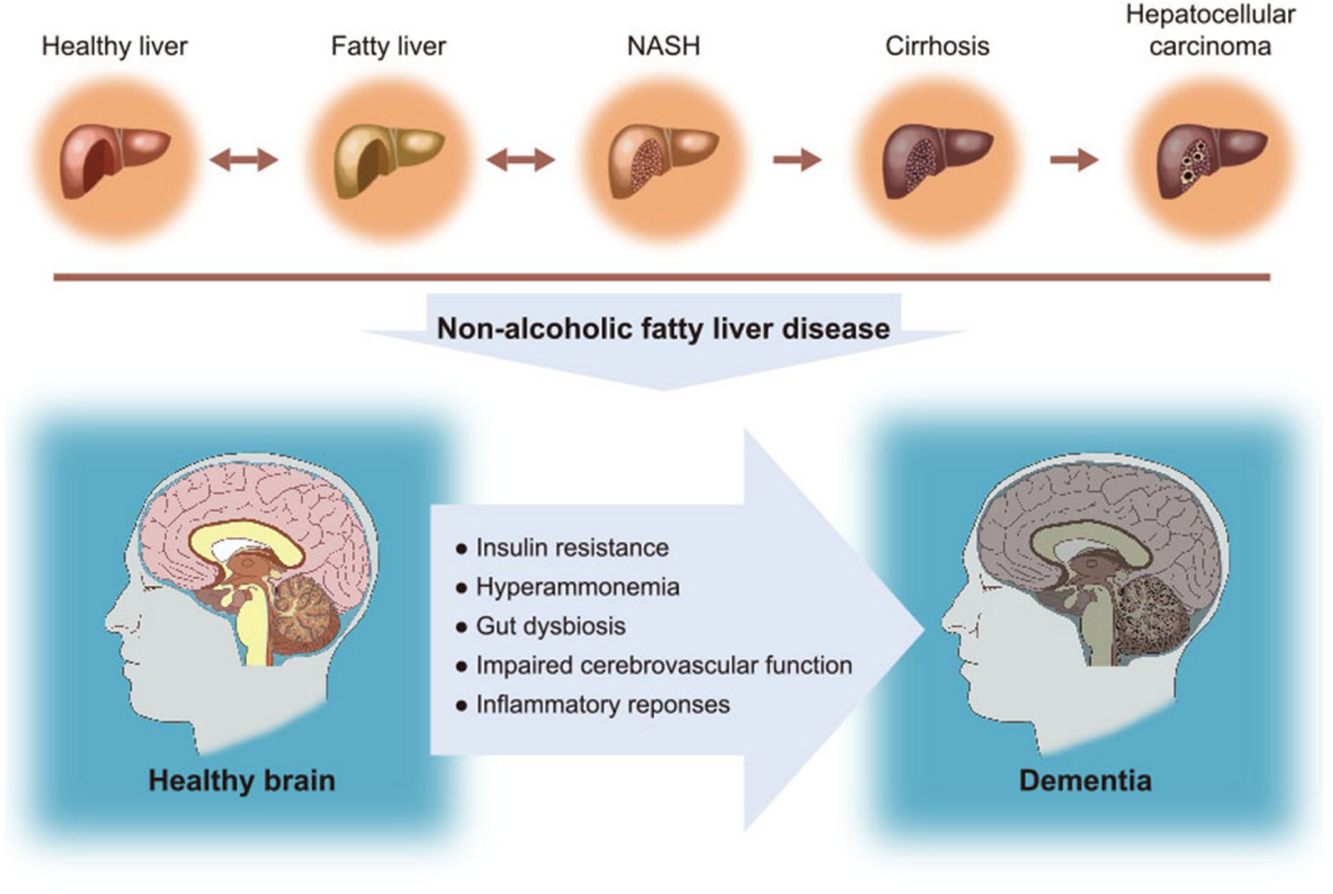
NAFLD as an important disease associated with the development of dementia. NAFLD is an important disease related to the development of dementia. It is associated with insulin resistance, hyperammonemia, gut dysbiosis, impaired cerebrovascular function, and inflammatory responses, leading to dementia

Recently, NAFLD has emerged as an important disease associated with the development of cognitive impairment and dementia [7, 11] (Fig. 1). Insulin resistance [12], hyperammonemia [13], vascular dysfunction, disruption of the gut microbiota, and inflammation [14], which are observed in patients with NAFLD, may be involved in neurological problems, such as cognitive impairment and memory loss [15–17]. For instance, NAFLD leads to cognitive impairment through insulin resistance and inflammation accompanied by excessive cytokine secretion [18]. The combination of hyperammonemia and inflammation induces cognitive dysfunction in patients with liver disease [19]. Even without liver disease, the interaction between hyperammonemia and inflammatory response results in cognitive impairment [19]. Furthermore, some studies have reported that patients with NAFLD have vascular dysfunction and impairment, such as decreased cerebral blood oxygen supply [20] and an alteration in the middle cerebral arteries [21], which can induce cognitive impairment [22]. Microbial dysbiosis is also positively correlated with recurrent hepatic encephalopathy (HE), and microbiota transplantation results in improved symptoms of HE [23].

Although many studies have investigated the association of NAFLD with cognitive dysfunction, the relationship between NAFLD and dementia remains to be completely elucidated. In this review, we summarize the recent evidence on the association between NAFLD and dementia with respect to common risk factors and pathologies.

### NAFLD and dementia

NAFLD is diagnosed based on imaging or liver biopsy assessments [24]. Additionally, changes in serum liver enzyme activity, such as altered serum alanine aminotransferase (ALT) and aspartate aminotransferase (AST) levels indicate abnormal liver function [24]. Liver function abnormalities negatively affect energy metabolism because the liver plays an key role in metabolism, and abnormal energy metabolism contributes to abnormal energy storage [24]. A variety of metabolic processes, such as glycolysis, lipogenesis, and gluconeogenesis are impaired, which ultimately leads to systemic metabolic disturbances, including insulin resistance and elevated serum free fatty acid and serum pyruvate concentrations [25]. Circulation of these metabolites can affect the brain [26]. Metabolic diseases such as NAFLD are shown to be significantly associated with an increased risk of dementia [27]. The pathogenetic mechanism underlying dementia is complex; dementia may occur as a complication of multiple diseases [28], and accurate history taking is important. Notably, NAFLD and dementia are shown to share many risk factors. In contrast, some studies have shown no association between NAFLD and cognitive impairment or between NAFLD and dementia [29, 30]. For example, a study has reported that diabetes or hypertension accompanied by chronic liver disease was not associated with cognitive impairment [29]. Patients with NAFLD who underwent liver biopsy did not show an increased risk of dementia [30]. Despite these reports, patients with NAFLD have an approximately four times greater risk of experiencing cognitive dysfunctions than control individuals [31]. Patients with NAFLD aged over 60 years exhibited lower cognitive function than did individuals of the same age without NAFLD [32]. A recent nationwide cohort study suggested an association between NAFLD and the risk of dementia [33]. A systematic review reported that patients with NAFLD showed a tendency to develop a decline in cognitive function and that multiple cognitive domains associated with general cognitive function, mental speed, attention, and mental flexibility were affected [34]. Anatomically, NAFLD manifests abnormal white matter integrity, reduced cerebral brain volume, and aberrant vascular changes leading to poor cognitive performance, which is considered the main feature of dementia [31, 35]. Magnetic resonance imaging (MRI) technique has revealed that patients with NAFLD display total brain atrophy [35], while a near-infrared spectroscopy tool has shown that the brain activity is decreased in these patients [20]. Similarly, Patient with NAFLD showed lower total cerebral blood flow and total brain tissue volume on MRI [36], difficulty in performing daily living activities [37], and lower cognitive function in the Symbol-Digit Substitution Test and Serial Digit Learning Test [38]. Visuospatial and executive dysfunctions were also observed [39]. NAFLD is associated with mental symptoms, such as anxiety and depression [40, 41]. An NAFLD animal model displayed neuronal loss in the frontal cortex [42], while a high-fat-diet-induced NAFLD model showed dopaminergic neuronal damage [43]. Similarly, studies using NAFLD animal models have reported changes in synaptic plasticity, which eventually led to cognitive dysfunction in the experimental animals [44]. Further, an NASH animal model showed decreased metabolic activities in multiple brain regions, such as the hippocampus, prefrontal cortex, thalamus, and amygdala [45], as well as cognitive deficits, including an impairment in social recognition and spatial working memory [45]. Hence, many studies have shown the association between NAFLD and cognitive deficits.

### Dementia

**Fig. 2:**
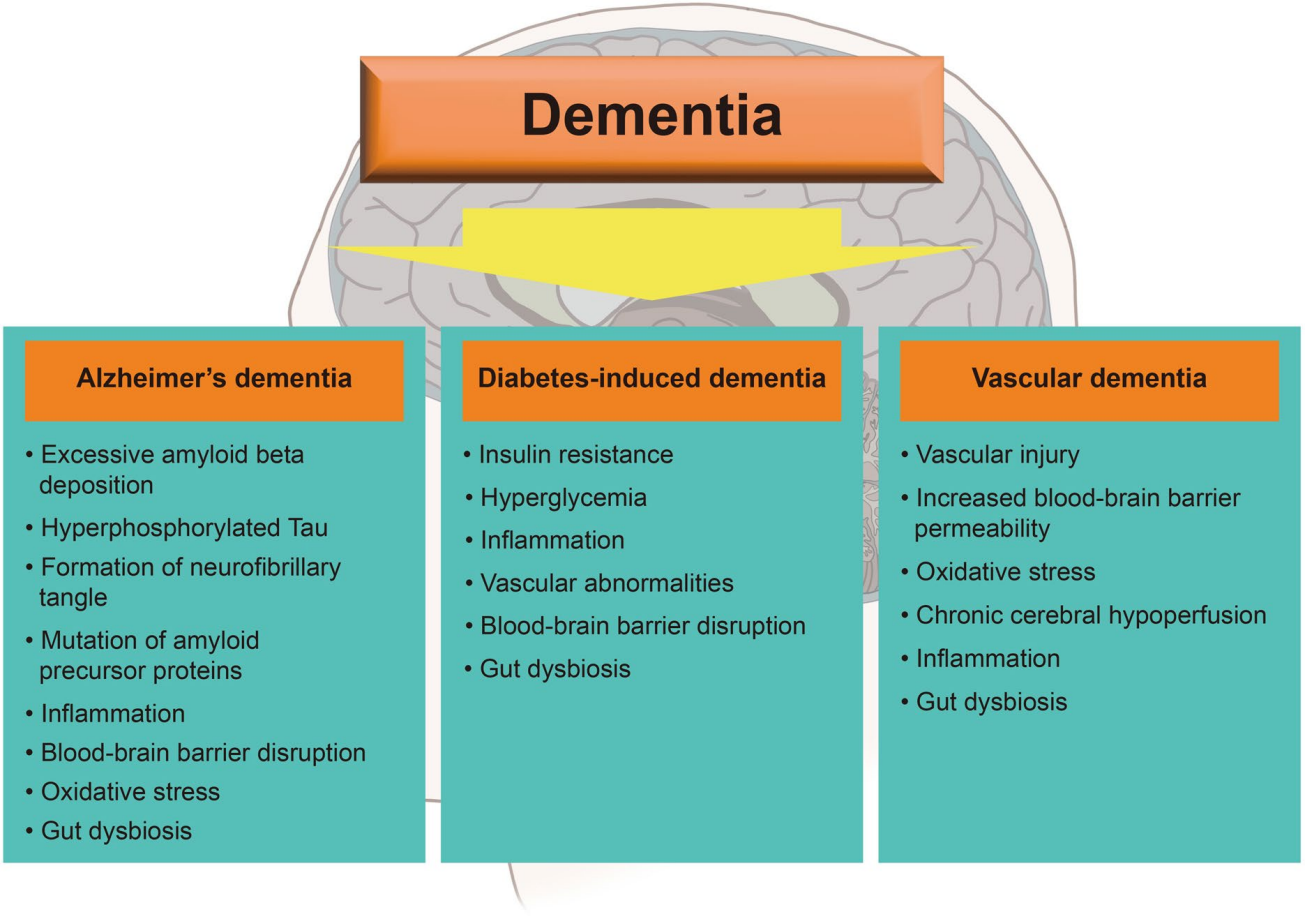
Common pathological features of dementia: Alzheimer’s dementia, diabetes-induced dementia, and vascular dementia. Dementia is classified into various types, including Alzheimer’s disease, diabetes mellitus-induced dementia, and vascular dementia. The major features are slightly different between the dementia types

Dementia is a common health problem worldwide that affects approximately 50 million individuals, and this number is expected to increase every year [46]. It is characterized by mental and cognitive degeneration, which results in progressive memory and cognitive loss [47]. Its subtypes are Alzheimer’s disease (AD), Lewy body dementia, vascular dementia, and frontotemporal dementia [47]. All dementia types are influenced by various risk factors. We summarize the characteristics of these dementia types below before discussing the relationship between dementia and NAFLD (Fig. 2). In this section, we attempted to explain how NAFLD is related to the risk factors for dementia.

#### Alzheimer’s dementia

AD is the most common type of dementia [48]. According to a recent report, Alzheimer’s dementia progresses slowly and affects an estimated 6.2 million individuals aged ≥ 65 years in the United States [49]. In addition, the susceptibility to AD is affected by sex differences, aging, environmental risk factors, and lifestyle [50]. The typical hallmark symptoms of AD are memory loss and impairment in new learning ability [51]. Patients with AD show attention and working memory impairments [51] as well as general cognitive impairments, including language and problem-solving difficulties [49]. Moreover, AD brains show poor short-term plasticity and long-term potentiation (LTP) processes compared with normal brains [52].

The common pathological features of AD are mutation of the amyloid precursor gene (APP), excessive amyloid beta deposition, senile plaque and neurofibrillary tangle formation, tau protein hyperphosphorylation, oxidative stress, blood–brain barrier (BBB) disruption, neuroinflammation, and gut dysbiosis [53–58] (Fig. 2). The onset and development of AD are affected by mutations in several genes, such as the *APP*, presenilin 1 (*PSEN1*), and *ApoE-ε4* [55]. Deposition of amyloid beta (Aβ_42_) plaque due to impairment of the clearance system is found in AD brains, which can induce hyperphosphorylated tau and tau deposition, resulting in neuritic dystrophy [53]. AD brains exhibit mitochondrial dysfunction, such as oxidative stress caused by reactive oxygen species (ROS) accumulation [56]. Changes in vascular territories, such as loss of vascular integrity, vascular malfunction, and BBB disruption, are observed in patients with AD [57]. AD brains also exhibit increased neuroinflammation with synaptic loss [59], poor inflammasome complex assembly due to lysosome disruption [60], hypometabolism, and microglial activation [61]. Moreover, an increase in the production of harmful substances owing to changes in the gut microbiota composition is associated with AD pathology [58].

Many studies have proven that a positive correlation exists between NAFLD and AD [62–64]. In protein–protein interaction analyses, NAFLD and AD share common genes and pathways [e.g., long-chain fatty acid signaling pathway, carbohydrate metabolism signaling pathway, interleukin (IL)-6, serine/threonine kinase 1 (AKT1), and vascular endothelial growth factor A] [62]. NLFLD increases the incidence of AD by triggering liver inflammation, neuroinflammation, and neuronal cell death [63]. Liver dysfunction observed in NAFLD reduces the hepatic expression of low-density lipoprotein (LDL) receptor-related protein 1, which is important for the clearance of circulating amyloid beta protein [64]. Similarly, hepatic dysfunction contributes to reduced clearance of peripheral amyloid beta [65]. AD with NAFLD displays several genes related to inflammatory response, senescence, and oxidative stress, which are likely to induce vascular dysfunctions and cerebral hypoperfusion [66]. In addition, AD patients with NAFLD show cognitive dysfunctions, including deficits in spatial working memory [66]. Liver enzymes are associated with the progression of AD [67]. In a previous cohort study, lower ALT levels resulted in elevated amyloid beta deposition, decreased cerebral glucose metabolism, and brain atrophy, ultimately leading to cognitive impairments [67]. Taken together, AD shares multiple pathological mechanistic processes and pathological factors with NAFLD.

#### Diabetes induced dementia

Diabetes mellitus (DM), particularly T2DM, is a common chronic metabolic disease worldwide, characterized by hyperglycemia and insulin resistance [68]. Because global metabolic dysfunction causes DM, patients with DM have higher risks for other metabolic diseases, such as cardiovascular, renal, and central nervous system (CNS) diseases [69]. Metabolic disturbances, such as DM and obesity, increase the risk for mild cognitive impairment and dementia [70, 71]. Recent studies also suggest that DM is a crucial risk factor for mild cognitive impairment or dementia [72, 73]. For this reason, diabetes-induced dementia is also called type 3 DM [74]. In particular, the prevalence of DM and dementia in elderly population is increasing [75], and patients with DM have an approximately 50% higher risk of developing dementia [76]. Patients with T2DM display brain changes, such as brain atrophy and cerebral lesions, on MRI [76]. They also show morphological changes, including reduced total white and gray matter and hippocampus volume, accompanied by impaired planning, visual memory, and visuospatial construction [77].

The pathological factors of diabetes, particularly T2DM, are insulin resistance, tau hyperphosphorylation, hyperglycemia, vascular dysfunction, inflammatory responses, BBB breakdown, and gut dysbiosis [78–82] (Fig. 2). Insulin resistance, defined as poor insulin sensitivity and deficient insulin signaling, is a well-known risk factor for DM [78]. Abnormally increased hyperphosphorylation of tau protein has been observed in DM [79]. Impaired insulin action contributes to poor glucose metabolism, neuronal damage, and impaired neurotransmitter secretion in the brain [83, 84]. Brain insulin resistance contributes to impaired hippocampal synaptic plasticity and impaired learning and memory function [85]. Insulin signaling results in hyperphosphorylation of tau through activation of glycogen synthase kinase 3 beta (GSK-3β) [86]. As a T2DM model, mice with high-fat-diet-induced hyperglycemia showed reduced learning and memory function [87]. Notably, high serum glucose levels, which are commonly observed in patients with diabetes, result in serious complications [88]. Vascular endothelial cells are highly vulnerable to glucose toxicity [88]. Hyperglycemia contributes to abnormal replication, aberrant cell cycle progression, and cell death in vascular endothelial cells, which can result in functional and structural abnormalities [89]. Furthermore, hyperglycemia alters the distribution of tight junction proteins and transporters of nutrients across the BBB, which compromises BBB permeability and function [90]. Therefore, patients with diabetes tend to develop vascular complications including retinopathy and neuropathy [88]. In addition, patients with T2DM exhibit increased oxidized LDL and C-reactive protein levels, which indicate oxidative stress and inflammation [80]. This interplay between oxidative stress and inflammation contributes to endothelial dysfunction [80]. MRI reveals the increased permeability of the BBB in patients with T2DM [91]. Additionally, the genera of *Blautia*, *Fusobacterium*, and *Ruminococcus* have been found in patients with T2DM, which have an impact on gut permeability, inflammatory response, and glucose metabolism [82].

A growing number of studies have reported that NAFLD is linked with DM [92, 93]. The conditions of patients with T2DM (70–80%) and type 1 DM (T1DM) (30–40%) are influenced by NAFLD [93]. Conversely, T2DM is also a risk factor for NAFLD to develop NASH to cirrhosis [92]. NAFLD contributes to insulin resistance, lipid dysmetabolism, and systemic inflammation, which also lead to T2DM [92]. NAFLD is closely related to macrovascular and microvascular complications observed in DM [93]. In T1DM and T2DM mice models, hyperglycemia promotes memory loss via high BBB permeability and microvessel dysfunction due to inflammation [94]. In gut microbial composition analyses, *Enterobacter*, *Romboutsia*, and *Clostridium *sensu stricto are relatively abundant in patients with T2DM during NAFLD progression [95]. Importantly, patients with both NAFLD and T2DM show cognitive impairments, including poor working memory or attention and delayed processing speed [96]. The combination of gamma-glutamyltransferase (γ-GT), whose levels are elevated in NAFLD and DM, results in the development of dementia [97]. Based on previous findings, DM and NAFLD share various pathological factors.

#### Vascular dementia

Vascular dementia is the second most common type of dementia and is characterized by global cognitive dysfunction caused by altered vascular factors and vascular injury [98, 99]. It can be classified into four major subtypes: poststroke dementia, mixed dementia, multi-infarct dementia, and subcortical ischemic vascular dementia [100]. Vascular pathologies, including cerebral microbleeds, atherosclerosis, amyloid angiopathy, and vessel disease, cause mild cognitive dysfunction and dementia [98, 101]. Patients with preclinical vascular dementia display lacunar infarcts detected in brain regions, including the striatum, internal capsule, and corona radiata, which govern language and memory [102]. Moreover, loss of gray matter density in the left hippocampus and right posterior putamen is observed in these patients [102]. Vascular brain injury can contribute to loss of brain connectivity, leading to impaired functional network in the brain [103]. Therefore, a large infarct volume and many small cortical infarctions are closely related to poor cognitive performance [103]. Patients with poststroke dementia display progressive decline of cognitive functions, impairments of executive functions, and delays in the processing speed [103].

The risk factors of vascular cognitive impairment, ranging from mild cognitive dysfunction to dementia, are vascular injury, chronic hypoperfusion, hypertension, hyperglycemia, BBB permeability, inflammation, and gut dysbiosis [103–107] (Fig. 2). Vascular damage, leading to vascular dementia, is known to result in brain endothelial dysfunction, increased BBB permeability, excessive ROS production, and hypertension [108]. Brain endothelial cell dysfunction, chronic hypoperfusion, BBB disruption, and impaired cerebrovascular reactivity lead to white matter lesions [109]. In addition, metabolic disturbances, such as DM and hyperglycemia, are risk factors for vascular dementia; therefore, patients with DM have a 2.4-fold higher risk for vascular dementia than individuals without DM [110]. Based on previous evidence, vascular cognitive impairment may be accompanied by an inflammatory process with BBB leakage [107], and the composition of the gut microbiota can induce inflammatory responses and increase the permeability of the intestinal epithelium, which can lead to the progression of vascular cognitive impairment [105]. Further, *Helicobacter pylori*-positive patients with vascular dementia display increased levels of inflammatory markers compared with *Helicobacter pylori*-negative patients with vascular dementia [106].

NAFLD is positively correlated with vascular dementia [35]. Patients with NAFLD have a high prevalence of all types of dementia, particularly, vascular dementia [97]. As aforementioned above, NAFLD is related to metabolic disturbances, such as DM and atherosclerosis, and affected patients show reduced blood flow, vascular structural and functional changes, including endothelial dysfunction, and vascular injury, as observed in vascular dementia [35]. Also, patients with NAFLD also have an increased risk for hypertension, similar to patients with vascular dementia [36]. These findings demonstrate the close relationship between NAFLD and vascular dementia.

### Insulin resistance in NAFLD and dementia

Insulin is a peptide hormone generated from the precursor proinsulin through various processes by the beta cells of the pancreatic islets [111]. It regulates energy metabolism by controlling glucose uptake in the liver, fat, brain, and muscle cells by binding with insulin receptors, such as insulin-like growth factor 1 (IGF-1) [112]. Insulin receptors are found in diverse brain regions, such as the hippocampus, hypothalamus, and cerebral cortex [112]. Many molecular signaling pathways are linked to insulin signaling pathways, such as the IGF-1/IGF-1 receptor-induced phosphoinositide 3-kinase (PI3K)/Akt signaling pathway, affecting axon development and synaptic formation [113]. Insulin can influence the hippocampal synaptic plasticity and memory formation by modulating LTP [113]. However, insulin resistance is defined as reduced insulin sensitivity [114] and brain insulin resistance as a failure of neurons and glia to respond to insulin [115]. The lack of insulin sensitivity arises from the reduced expression of insulin receptors, inability of the insulin receptor to bind insulin, and impairment of insulin signaling [112, 115]. Insulin resistance causes imbalanced secretion of neurotransmitters, such as acetylcholine, as well as impaired synapse remodeling, poor memory formation, and cognitive dysfunction [113, 116, 117].

Insulin resistance is considered a common risk factor for NAFLD and dementia [27, 118] (Fig. 1). Insulin resistance and poor insulin sensitivity are hallmark features of T2DM and NAFLD [119]. A defect in insulin signaling causes intrahepatocellular lipid accumulation and altered free fatty acid degradation by the hepatic pathway, leading to NAFLD [120]. Insulin resistance is an important sign for the progression of NAFLD including NASH [121]. NAFLD is linked to insulin resistance and obesity and that DM tends to coexist with NAFLD [122]. Insulin resistance in patients with obesity and T2DM ultimately leads to liver dysfunction and contributes to the development of NAFLD [118]. Brain insulin deficits and hyperinsulinemia are known to lead to memory loss and the development of dementia [123].

According to a large population follow-up clinical study, a triglyceride glucose index is positively related to high probabilities of dementia progression, including vascular dementia or AD [124]. In addition, a previous study has reported that the apolipoprotein E alleles, *ApoE-ε4* allele, a crucial risk factor for the development of dementia [125], was detected in both patients with DM and patients with AD [83]. The presence of the *ApoE-ε4* allele increases the risk of brain insulin resistance, impaired glucose metabolism, and tau hyperphosphorylation [83, 126].

Given these findings on the relationship between insulin resistance, NAFLD, and dementia, insulin resistance may be a therapeutic target for both NAFLD and dementia, for which insulin resistance is considered an important trigger [27, 118, 127]. The modulation of insulin resistance may simultaneously suppress the risk of NAFLD development and dementia progression [128–130].

### Inflammation in NAFLD and dementia

Inflammation is a common characteristic of patients with NAFLD, which is considered a chronic inflammatory disease [131]. After inflammatory injury, circulating immune cells secrete excessive proinflammatory chemokines and cytokines and transmigrate into the brain through the BBB [132]. The infiltrated immune cells activate periventricular resident microglia near the blood vessels [132]. The interplay between the recruited and resident immune cells results in neuroinflammation, subsequently leading to neurological diseases [132]. A previous study has shown that inflammatory liver injury-induced hepatic inflammation drives cerebral inflammation and sickness behavior [132]. Neuroinflammation can impair axons and myelin sheaths, induce abnormal levels of inflammatory cytokines, including tumor necrosis factor-α, IL-1β, and IL-6, and lead to neurotoxic effects [133]. Although immune cells are important for tissue regeneration and repair [134], chronic inflammatory responses result in mental illness, cognitive impairment, and dementia [135, 136].

Inflammation is positively correlated with NAFLD and dementia [136–139]. In NAFLD, the excessive production of proinflammatory cytokines and chemokines is triggered by hepatic fat accumulation and hepatocyte damage [137]. Excessive hepatic lipid accumulation triggers macrophage activation, hepatic inflammation, and liver fibrogenesis [137]. Subsequently, this excessive secretion of proinflammatory mediators accelerates the progression of NAFLD into NASH [137]. Increased liver inflammation in NAFLD induces microglia activation and neuronal cell death, ultimately resulting in AD dementia, indicating that systemic inflammation in NAFLD contributes to the progression of severe cognitive dysfunction [63]. Moreover, the activation of the nuclear factor-kappa B (NF-κB) pathway, an inflammatory regulatory signaling pathway, leads to the chronic secretion of proinflammatory cytokines and impairment of both hepatic and systemic insulin sensitivities [140]. Immune cells, such as macrophages and natural killer T cells, induce the progression of NAFLD [137, 141]. Chronic neuroinflammation by the activated microglia and cytokines is observed in the brain of patients with AD [136], and elevated systemic inflammation results in increased risk for diabetes dementia [139].

Thereby, chronic or systemic inflammation induces the secretion of inflammatory cytokines in the microglia and immune cells and ultimately the development of both NAFLD and dementia [136, 137, 139]. The modulation of systemic inflammation and neuroinflammation is an important strategy in attenuating the pathological problems associated with NAFLD and dementia.

### Hyperammonemia in NAFLD and dementia

Ammonia is the end-product of body metabolism; however, high ammonia levels are neurotoxic [142, 143]. Cell death signaling pathways, including the NF-κB signaling pathway, apoptosis markers, nitric oxide activity, and superoxide in glial cells, are promoted by high ammonia levels [144, 145]. In the CNS, ammonia is known to cross the BBB and is controlled by astrocytes; thus, the ammonia level is important in the pathogenesis of liver diseases [146]. Hyperammonemia, the excessive accumulation of ammonia, causes liver damage and fibrosis and accelerates the progression of NASH [13]. In addition, it also aggravates the inflammatory response, brain edema, and cognitive impairment [143, 147].

Abnormal ammonia levels are detected in NAFLD and dementia [13, 142, 148]. NAFLD is characterized by urea cycle impairment, leading to a hyperammonemia state caused by an impaired ammonia–nitrogen conversion cycle [13]. Hyperammonemia triggers neuronal damage, astrocyte swelling, and poor synaptic plasticity, leading to memory loss in NAFLD [143, 149]. Further, hyperammonemia and neuropsychiatric problems, including personality, cognition, and consciousness problems, are observed in these patients [19]. A recent study reported that NASH is accompanied by hyperammonemia and imbalanced neurotransmitter secretion in the brain, leading to memory deficits [45]. Elevated ammonia levels and abnormal ammonia metabolism were observed in AD, and hyperammonemia was associated with the progression of AD [142, 148]. High levels of ammonia cause mitochondrial dysfunction, increased activity of poly (ADP-ribose) polymerase (considered a cell death index), and excessive ROS production in the AD brain [142]. Additionally, hyperammonemia increases the secretion of gamma-aminobutyric acid (GABA), an inhibitory neurotransmitter, and causes memory deficits in AD [142]. In AD, excessive deposits of amyloid beta alter the glutamate synthetase enzyme that detoxifies ammonia, and the resulting increase in the ammonia levels consequently results in a neurotoxic condition [150]. Increased release of ammonia is also observed in patients with T2DM [151].

Given these previous findings, elevated ammonia levels contribute to the progression to a more severe form of NAFLD, followed by memory loss, eventually leading to dementia. Controlling the ammonia level in the brain may be a good approach for both NAFLD and dementia.

### Gut dysbiosis and impaired gut barrier integrity in NAFLD and dementia

The gut microbiota controls multiple metabolic and physiological homeostasis processes in the body [152]. Nutrients can change the composition and function of the gut microbiota [152], as well as contribute to brain functioning and many mechanisms in the CNS [153]. The composition of the gut microbiota contributes to the regulation of hormone secretion, gene expression, neurotransmitter secretion, and immune function [154, 155]. The microbiota affects neuronal activity, neuronal gene expression, and synaptic dendritic spine remodeling [156]. Gut dysbiosis results in neurological diseases including depression, anxiety, and autism [157]. In addition, gut barrier homeostasis is maintained by various tight junction proteins within the epithelium and is an excellent barrier to bacteria and toxic metabolic products [158]. Increased gut permeability caused by damage to the intestinal barrier allows toxic metabolites and bacterial fragments to enter the liver and triggers hepatic inflammation and liver fibrogenesis [158, 159]. Gut permeability triggers systemic inflammation, followed by neuroinflammation, leading to cognitive dysfunction [160].

Gut dysbiosis is closely associated with NAFLD and dementia [161–164]. The brain–gut–liver axis is impaired in patients with NAFLD [165]. During the development of NAFLD, patients show gut microbiota changes, low bacterial diversity, and increased Firmicutes/Bacteroidetes ratio [161]. In patients with NAFLD, gut dysbiosis enhances insulin resistance and gut intestinal permeability, involving a chronic immune response [159, 166]. Similarly, a recent clinical study highlights that increased gut permeability and impaired gut homeostasis occur in metabolic disorders, such as NAFLD [167]. One study observed a leaky gut and found reduced ZO-1 tight junction proteins in the small intestines of patients with NAFLD [168]. Abundant numbers of Proteobacteria [169], *Bacteroides* [170], Gammaproteobacteria, and *Prevotella* [171] were found in patients with NAFLD compared with those in their normal counterparts. During the progression of liver failure, increased Proteobacteria phylum and decreased Firmicutes phylum were observed in patients with NAFLD [172]. Administration of probiotics enhances the gut–liver–brain axis and suppresses the development of NAFLD by reducing insulin resistance and, the levels of total cholesterol, ALT, AST, and inflammatory mediators [166]. The administration of a mixture of six probiotics or administration of *Akkermansia muciniphila* suppressed hepatic fat, increased the level of glucagon-like peptide 1 (GLP-1), and improved the gut barrier integrity, thereby leading to improved haptic status in patients with liver disease [173–175]. Administration of probiotics (*Lactobacillus plantarum*) improved cognitive impairment by modulating hippocampal TLR4/BDNF signaling in an NASH model [176]. Importantly, gut dysbiosis is associated with the development of dementia, and gut inflammation and reduced gut microbial diversity are strongly linked to AD [162–164]. One study found that the gene expression of amyloid beta protein affected the gut microbiome composition in an AD animal model [177]. Gut dysbiosis occurs in patients with dementia and causes dysregulation of anti-inflammatory pathways [178]. A reduced microbial diversity aggravates dyslipidemia, inflammation, and insulin resistance, leading to metabolic syndromes, such as DM and obesity [179, 180].

As mentioned above, gut dysbiosis and increased gut permeability are important factors in accelerating both NAFLD progression and cognitive dysfunction, leading to dementia. The manipulation of the gut microbiome may be beneficial in NAFLD and dementia. The finding of common microbiome species between NAFLD and dementia may provide a basis for developing treatments for the pathologies associated with these two diseases.

### Impaired cerebrovascular function in NAFLD and dementia

The neurovascular unit is formed by diverse cell types, such as astrocytes, endothelial cells, pericytes, smooth muscle cells, and neurons [181]. Neurovascular coupling is a structural and functional term related to neural activity and cerebrovascular blood flow [181]. Neurovascular decoupling accelerates the development of neurodegeneration by inducing brain dysfunction/injury and promoting the release of various metabolites and chemical mediators [181]. Vascular disturbance results in chronic hypoperfusion, BBB disruption, neurotoxic molecule accumulations, and amyloid beta accumulation, ultimately leading to dementia and AD [182].

Vascular abnormalities are risk factors for the progression of NAFLD and dementia [183, 184]. Previous studies have demonstrated that NAFLD is associated with vascular disease risk factors, cerebrovascular and cardiovascular dysfunctions, and increased risk for DM and hypertension [183]. NAFLD is affected by vascular complications and triggers hypertension and atherosclerosis [185, 186]. It causes CVD by increasing the carotid intima–media thickness, arterial stiffness, and coronary artery calcification [187]. Patients with NAFLD had a reduced cerebral blood flow in the middle cerebral artery [21]. Cerebrovascular alterations due to vascular deterioration accelerate the progression of NAFLD and cognitive deficits by changing the brain structure [36]. NAFLD decreases the total cerebral blood flow, induces microvascular alteration, and finally triggers cognitive dysfunction [36]. Similarly, another recent study found that patients with NAFLD simultaneously show cerebrovascular dysfunction and memory loss [32]. Neurovascular dysfunctions, such as neurovascular decoupling and abnormal cerebral blood flow, lead to AD [188]. Apolipoprotein E4 (*APOE4*), which is associated with atherosclerosis and coronary heart disease, is strongly related to an increased risk for AD development [189]. Cerebral hypoperfusion and vascular abnormalities contribute to cognitive dysfunction and AD, and patients with AD or vascular dementia show cerebrovascular lesions [190, 191]. Microvascular and macrovascular abnormalities, including vascular death, are very common in diabetes and vascular dementia [98, 101, 192].

Given these relationships between NAFLD and vascular complications, modulation of the cerebrovascular system is needed to treat NAFLD and dementia. Controlling the vascular risk factors may be a good approach in the treatment of NAFLD and dementia.

## Conclusions

**Fig. 3:**
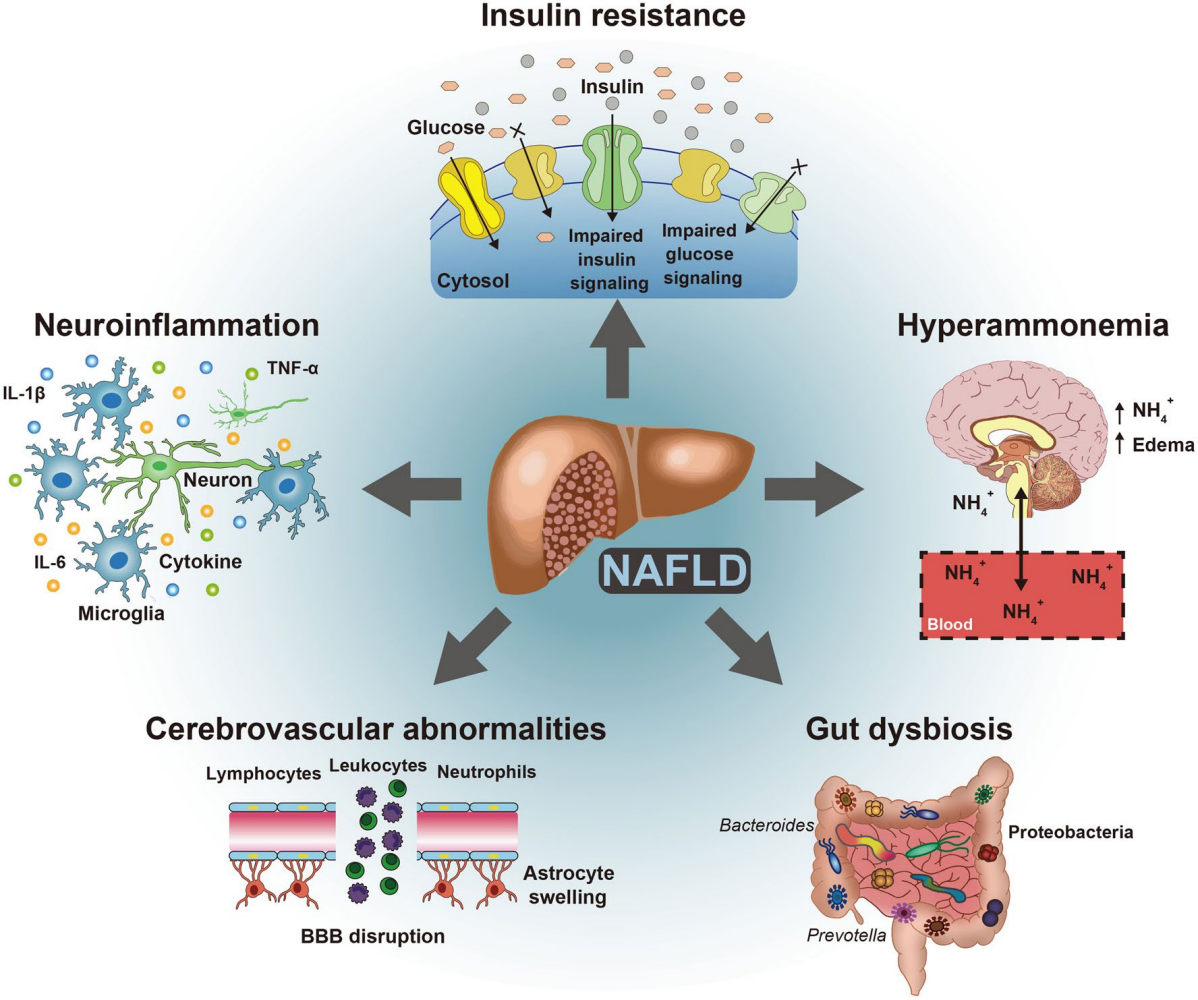
NAFLD-caused symptoms, including insulin resistance, neuroinflammation, hyperammonemia, gut dysbiosis, and cerebrovascular dysfunction, associated with some dementia. NAFLD can lead to chronic neuroinflammation, brain insulin resistance, hyperammonemia, cerebrovascular abnormalities, and gut dysbiosis. Those symptoms are closely associated with some dementia

In this review, we summarized the dementia-related pathological features of NAFLD, such as insulin resistance, neuroinflammation, hyperammonemia, gut dysbiosis, and cerebrovascular dysfunction (Fig. 3). Insulin resistance is a common risk factor for both NAFLD and dementia, displaying increased insulin signaling-mediated cell death, impaired LTP, and imbalanced neurotransmitter secretion. Systemic inflammation triggers macrophage secretion of inflammatory cytokines, induces immune cell infiltration and microglia activation, attenuates synapse formation, and increases neuronal cell death. Hyperammonemia results in impaired urea cycle and contributes to astrocyte swelling, BBB disruption, abnormal energy cycle, excessive ROS production, neuronal cell death, and increased inhibitory neurotransmitter GABA secretion. Gut dysbiosis and increased gut permeability enhance insulin resistance and inflammatory response and reduce the GLP-1 level. Impaired cerebrovascular function contributes to BBB disruption, neurotoxic molecule accumulations, excessive amyloid beta accumulation, cerebrovascular blood flow disruption, microvascular infarction, and brain atrophy.

Many etiological factors are known to contribute; however, a limited number of prescription drugs are approved for dementia by the Food and Drug Administration (FDA) [193]. Moreover, owing to the high prevalence of Alzheimer’s dementia [193], pharmacological management of dementia (using acetylcholinesterase inhibitors, N-methyl-d-aspartate [NMDA] receptor antagonists, and combination treatment) is primarily focused on Alzheimer’s dementia, and this approach may be ineffective in treating or delaying symptoms of other types of dementia. Several trials have shown that cholinesterase inhibitor therapy is beneficial for the management of patients with vascular cognitive impairment [194], and memantine (an NMDA receptor antagonist) administration improves cognitive function in patients with mild-to-moderate vascular dementia [195]. However, concomitant adverse effects such as dizziness, headache, and nausea have been reported [194, 196], and these should not be overlooked. In view of an increase in the number of patients with NAFLD and the prevalence of dementia in patients with NAFLD, further studies are warranted to investigate and gain a deeper understanding of the association between NAFLD and dementia. For those reasons, this review provides new perspectives on cognitive impairment in patients with NAFLD and suggests potential strategies for treating cognitive impairment in such patients.

## Data Availability

Not applicable.

## Abbreviations

NAFLD: Non-alcoholic fatty liver disease
NAFL: Non-alcoholic fatty liver
NASH: Non-alcoholic steatohepatitis
CKD: Chronic kidney disease
CVD: Cardiovascular disease
MRI: Magnetic resonance imaging
APP: Amyloid precursor gene
AKT1: Serine/threonine kinase 1
VEGFA: Vascular endothelial growth factor A
LRP-1: Low-density lipoprotein receptor-related protein 1
GSK-3β: Glycogen synthase kinase 3 beta
LDL: Low-density lipoprotein
γ-GT: Gamma-glutamyltansferase
IGF-1: Insulin-like growth factor 1
TNF: Tumor necrosis factor
APOE4: Apolipoprotein E4
LTP: Long-term potentiation
AD: Alzheimer’s disease
CNS: Central nervous system
T2DM: Type 2 diabetes mellitus
ROS: Reactive oxygen species
BBB: Blood–brain barrier
DM: Diabetes mellitus
MAPK: Mitogen-activated protein kinase
NF-κB: Nuclear factor-kappa B
IL: Interleukin
GABA: Gamma-aminobutyric acid
GLP-1: Glucagon like peptide 1
